# A Clinical Study to Assess Diffuse Reflectance Spectroscopy with an Auto-Calibrated, Pressure-Sensing Optical Probe in Head and Neck Cancer

**DOI:** 10.3390/curroncol30030208

**Published:** 2023-02-24

**Authors:** Ashlyn G. Rickard, Husam Mikati, Antoine Mansourati, Daniel Stevenson, Marlee Krieger, Daniel Rocke, Ramon Esclamado, Mark W. Dewhirst, Nirmala Ramanujam, Walter T. Lee, Gregory M. Palmer

**Affiliations:** 1 Department of Radiation Oncology, Duke University Medical Center, Box 3455, Durham, NC 27710, USA; 2 Zenalux Biomedical, Inc., Durham, NC 27705, USA; 3 Department of Biomedical Engineering, Duke University Medical Center, Box 3455, Durham, NC 27710, USA; 4 Department of Head and Neck Surgery & Communications Sciences, Duke University Medical Center, Box 3455, Durham, NC 27710, USA

**Keywords:** diffuse optical spectroscopy, head and neck squamous cell carcinoma, diffuse reflectance spectroscopy, optical biopsy, optical sensing

## Abstract

Diffuse reflectance spectroscopy (DRS) is a powerful tool for quantifying optical and physiological tissue properties such as hemoglobin oxygen saturation and vascularity. DRS is increasingly used clinically for distinguishing cancerous lesions from normal tissue. However, its widespread clinical acceptance is still limited due to uncontrolled probe–tissue interface pressure that influences reproducibility and introduces operator-dependent results. In this clinical study, we assessed and validated a pressure-sensing and automatic self-calibration DRS in patients with suspected head and neck squamous cell carcinoma (HNSCC). The clinical study enrolled nineteen patients undergoing HNSCC surgical biopsy procedures. Patients consented to evaluation of this improved DRS system during surgery. For each patient, we obtained 10 repeated measurements on one tumor site and one distant normal location. Using a Monte Carlo-based model, we extracted the hemoglobin saturation data along with total hemoglobin content and scattering properties. A total of twelve cancer tissue samples from HNSCC patients and fourteen normal tissues were analyzed. A linear mixed effects model tested for significance between repeated measurements and compared tumor versus normal tissue. These results demonstrate that cancerous tissues have a significantly lower hemoglobin saturation compared to normal controls (*p* < 0.001), which may be reflective of tumor hypoxia. In addition, there were minimal changes over time upon probe placement and repeated measurement, indicating that the pressure-induced changes were minimal and repeated measurements did not differ significantly from the initial value. This study demonstrates the feasibility of conducting optical spectroscopy measurements on intact lesions prior to removal during HNSCC procedures, and established that this probe provides diagnostically-relevant physiologic information that may impact further treatment.

## 1. Introduction

There is a significant, unmet clinical need associated with screening, diagnosis and treatment of head and neck squamous cell carcinoma (HNSCC). In the United States alone, ~65,630 people (48,200 men and 17,430 women) have developed HNSCC in 2020, and 14,500 patients died [1]. Most HNSCC are located in the lining of the mouth, nose and throat [2,3], and—like other cancers—these are staged using the Tumor Nodes Metastasis (TNM) system which provides prognostic information and guides therapeutic decision making in newly diagnosed patients. However, the anatomically based TNM system faces significant challenges; it often fails to accurately describe the metastatic and invasive potential in early-stage lesions. For example, patients presenting with the same stage disease may exhibit markedly different outcomes despite receiving identical treatments based on initial TNM staging [4]. In the past several decades, researchers have sought to eliminate this unreliability by compounding TNM staging with functional biomarker measurements; these biomarkers further describe the lesion and, ideally, predict the best treatment approach in conjunction with the prognosis.

Identifying physiologic and biologic prognostic/predictive factors requires devices that exceed the current clinical standards of care. This tool requires (1) rapid feedback, (2) non-invasiveness, (3) portability, (4) a quantitative approach and (5) minimal operator bias. An instrument that meets these requirements could significantly decrease the emotional and physical toll of patients with suspicious lesions and prioritize patients with a high likelihood of malignancy by increasing clinical efficiency [5].

Diffuse reflectance spectroscopy (DRS) offers a non-invasive, non-destructive and quantitative approach for measuring vascular oxygenation and vascularity. This involves illuminating the tissue using a white light source and measuring the spectrum of diffusely reflected light with a fiber optic probe. DRS is an easily repeatable, in situ measurement, making it an ideal system for monitoring functional changes. In tumors, DRS has previously been used for measuring biological endpoints in squamous cell carcinoma, breast, cervix, HNSCC and other cancers [6,7,8,9]. Unlike more traditional methods for monitoring tumor vascularization and tissue characteristics, such as immunohistochemistry and optical imaging, DRS is used in vivo and achieves greater penetration depth as compared to ballistic optical imaging methods due to the use of diffusely scattered photons [10,11]. There has also been promising work with diffuse optical tomography, which is a related modality to DRS, where near-infrared images of the absorption and scattering data within tissue are acquired in combination with standard imaging modalities, particularly in breast cancers [12,13,14]. For applications in HNSCC, however, a smaller probe is better suited to obtaining functional measurements as long as it can be rigorously optimized as DRS is generally not used in combination with ultrasound, MRI or x-ray diagnostic imaging. Prior studies using DRS have found that reproducibility is improved with real-time assessment of probe pressure and system throughput calibration [5], which must be addressed before widespread clinical testing.

Calibration is required to compensate for the wavelength-dependent instrument response, lamp intensity fluctuations, and fiber bending losses [11,15]. However, there are several limitations associated with calibrating instruments, especially with different users. For instance, uncontrolled probe-to-tissue coupling (e.g., pressing too hard against the tissues) makes it difficult to produce a reproducible spectrum, particularly for an inexperienced operator. Recently, Ti and Lin, et al. [16] studied the short- and long-term effects of probe pressure in in vivo diffuse reflectance preclinical studies. They concluded that elevation in probe pressure induces major alterations in the profile of the reflectance spectra between 400 and 650 nm, and the changes in the tissue optical properties are dependent on probe pressure and tissue type.

Using in-depth and time-consuming calibration protocols, it has been demonstrated that measured oxygen saturation (SO_2_) in preclinical models can accurately predict tumor responses. Using DRS in a human head and neck cancer xenograft model (FaDu), SO_2_ was measured in the in the first two weeks after a single dose of radiation. It was possible to discriminate between complete responders (no evidence of tumor over a 120-day period) and partial responders (tumor persistence within a 120-day period) weeks before there were significant differences in tumor volume [17]. In clinical HNSCC studies, it has been shown that DRS can be used to extract SO_2_, THb (total hemoglobin concentration) and μ_s_′ (the reduced scattering coefficient that reflects tissue morphology and the presence of scattering structure such as fibrous tissue) [18,19]. Notably, the ability to differentiate between tissue types (from 112 normal and 36 squamous cell carcinoma samples) has been demonstrated using SO_2_, log(THb) and log(μ_s_′) measurements. These functional data were input into a logistic regression model; the receiver–operator curve had an area under the curve of 0.84 [18]. These studies indicate that DRS can accurately differentiate tissue types and offer both diagnostic and prognostic information. 

More recently, we have shown that pressure control and self-calibration can significantly reduce systematic errors in the measurement of THb and μ_s_′, respectively [5]. This was demonstrated in a pilot study with healthy volunteers. We evaluated the influence of pressure and found that by controlling and minimizing the probe pressure, it is possible to minimize pressure-induced changes that occur in the tissue being measured [5].

With improved self-calibration and pressure-sensing capabilities, we expect that robust, precise, accurate and quantitative measurements of tumor physiology will lead to significantly reduced errors in the extracted parameters [5]. Our goal was to measure tissue absorption and scattering difference between a “tumor” site and distant “normal” site in a pilot cohort of HNSCC patients and to correlate our results with gold-standard pathological results. This allows us to quantify differences in optical biomarkers from normal and cancerous sites. The long-term objective is to apply diffuse reflectance spectroscopy to enable real-time diagnosis of HSNCC. 

## 2. Materials and Methods

### 2.1. Clinical Study

Nineteen HSNCC patients scheduled for either/or panendoscopy and/or biopsy were part of this clinical optical evaluation study. The Institutional Review Board at Duke University approved this study, under Protocol number: Pro00065967. Before study participation, all patients provided written, informed consent to non-invasive evaluation by optical probe of biopsy sites. These sites included those identified as “tumor,” and “distant normal”. Sites were all chosen based on the clinical judgement of the treating physicians who are all board-certified head and neck surgeons. Where multiple independent tumor lesions were present, additional tumor sites were sampled as time allowed (two patients had multiple lesions included). All “distant normal” measurements were assumed to be normal. Five optical measurements were repeated at each site to establish measurement consistency. There were 8 tumor sites on the tongue, 1 site on the larynx, 1 site on the oropharynx, 1 site on a tonsil and 1 site on a piriform lesion. There was an additional measurement made for a patient with benign atypical verrucous hyperplasia. A biopsy was taken from each tumor site for histopathological diagnosis to be compared with Zenascope measurements. Patient characteristics are summarized in Table 1.

### 2.2. Optical Spectroscopy

**Figure 1:**
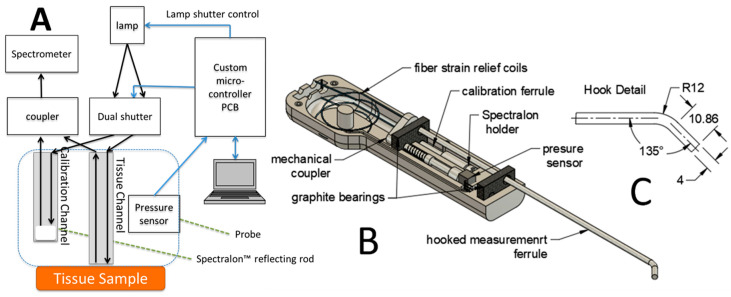
An instrument schematic shows the system components. (**A**) The instrument includes an optical switch (dual shutter) to direct light to either a calibration or tissue-sensing channel for automated calibration measurement. (**B**) The probe handle (internals exposed) features a pressure-sensing system that uses a spring-loaded metal ferrule that contacts a force sensor. The probe ferrules were uncoupled from the weight of the cable through use of a coiled segment of bare cable that can flex as the fiber tip is pressed against the sample. (**C**) An engineering drawing illustrating dimensions (mm) of the hooked sample ferrule. Straight ferrule not shown.

The diffuse optical spectroscopy system Zenascope PC2 (Zenalux Biomedical Inc., Durham, NC, USA) was used for all measurements. The system consists of a console with a 40 W halogen lamp (HL2000HP; Ocean Optics, Dunedin, FL, USA) for a light source, spectrometers (USB2000; Ocean Optics), and 2 optical probe fibers (750 µm core diameter) for illumination and collection. Two different probe types were used depending on the measurement site: either a straight or hooked design (illustrated in Figure 1). The hooked design was generally used for deeper sites within the oral cavity while the straight probe was used for oral sites; the physician chose which would be more suitable. 

Automated pressure-sensing and repeated measurements were acquired in a single probe placement. The procedure for the pressure-sensing probe and auto-calibration in Zenascope have been described in recent papers [5]. Briefly, instead of calibration being performed at the beginning and end of the study, rapid measurements were taken immediately before each data point was acquired. The internal switch allowed for rapid switching between measurement and calibration channels. A series of 5 repeated measurements were taken from each site to evaluate any potential changes in DRS signal over time. 

### 2.3. Self-Calibration

Using previous systems, calibration has commonly been performed at the beginning or end of a study to account for instrument fluctuations related to lamp or power drift. Calibration had also typically required waiting for an additional 10–20 min for the lamp to warm up and stabilize. This was a significant amount of time in a clinical setting between spectrometer data and calibration channels. 

To address the above challenges, an automatic optical switch is incorporated into the device to enable the rapid switching (on the order of milliseconds) of the spectrometer between tissue measurement data and calibration channels (Figure 1A). This allows real-time calibration of lamp intensity and fluctuations due to fiber bending.

### 2.4. Pressure-Sensing Probe

The probe rests against a pressure sensor within the handle (Figure 1B) that monitors pressure while taking tissue measurements. This force sled and spring (schematically shown in Figure 1) allows a force to be transferred from the tissue to the force sensor; stability and consistency in the force are applied by reducing the slope of force vs. displacement without relying solely on tissue compliance. Linear motion micro positioners and pressure sensors with a feedback loop are integrated to maintain probe contact pressure rather than having the user control it manually. The pressure sensor was set to trigger a good measurement when the pressure was between 24 and 72 mmHg, which was previously found to provide consistent probe contact with minimal deviation in signal over time in a healthy volunteer study [5].

### 2.5. Spectral Data Analysis

**Figure 2:**
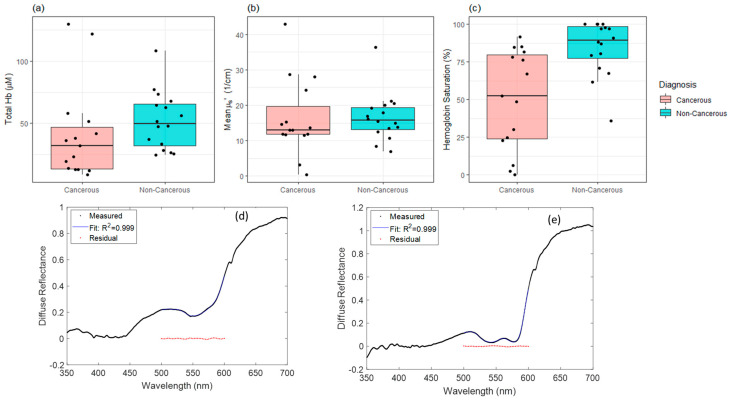
Data summary based on DRS evaluation using pressure-sensing and self-calibrated data. Samples are grouped together and plotted based on measurement sites that are cancerous and non-cancerous. The total hemoglobin (**a**) and mean reduced scattered (*µ_s_*′) (**b**), and the percent hemoglobin saturation (**c**) are shown. Hemoglobin saturation showed a significantly lower value for cancerous as compared to non-cancerous tissue type (*p* < 0.001). In addition, sample spectra including one cancerous SCC of the tongue (**d**), and one normal (**e**) tissue site (paired samples from the same patient). Also shown are the fitted spectra and residual plots, with fitting performed over the 500–600 nm wavelength range. In this case, a highly deoxygenated sample is indicated by the presence of only a single absorption band near 550 nm in the tumor sample (**d**), whereas two absorption bands are seen close to 540 and 580 nm in the well oxygenated normal sample (**e**), corresponding to the alpha and beta bands of oxyhemoglobin absorption. The fitted spectra can be difficult to distinguish from the measured spectra, but these largely overlap within the thickness of the plotted lines.

A quantitative inverse Monte Carlo model was used to extract scattering and absorption properties of the measured tissues [20]. To briefly summarize, this Monte Carlo model was implemented using custom Matlab and Python code and integrated into the system control software. The inverse model works by fitting the measured diffuse reflectance spectra using a non-linear least squares algorithm to match a Monte Carlo-based modeled spectrum. The optical properties are constrained using the known spectra of the absorbers and scatterers present in the tissue. An example of such a fit is shown in Figure 2. Using this inverse Monte Carlo model, concentrations of oxygenated hemoglobin (HbO_2_), deoxygenated hemoglobin (dHb), and total hemoglobin (THb) and oxygen saturation (SO_2_) were calculated in μM [21].

Diffuse reflectance spectra obtained from the tissue sample were sampled at 2.5 nm increments over a wavelength range from 500 to 600 nm. This provided sufficient data to perform the least squares fit. Background data were subtracted by measuring the sample with the lamp shutter closed. Data were also normalized to the Spectralon reflectance standard measurement in the calibration arm to obtain the diffuse reflectance relative to the spectrally flat standard.

The model assumed absorption could be characterized by a combination of oxy- and deoxy- hemoglobin, modified by the pigment packing factor of van Veen, et al. [21], and a non-melanin skin absorption component [22]. Scattering is approximated by the relationship

μ_s_′ = A × λ^−b^, (1)

where *μ_s_′* is the reduced scattering coefficient, *A* is the scattering amplitude, *λ* is the wavelength in nanometers and *b* is the scatter power factor. The model inputs, including absorber concentrations and scattering parameters, were adjusted to obtain a least-squares fit to the measured data using the Python SciPy library [23].

A multivariate linear mixed effects model was used to test for significant main effects for pressure (modeled as a factor), time (represented by repeated sample number and modeled as a continuous variable) and site (modeled as a factor). Both hemoglobin oxygen saturation and total hemoglobin content were used as response variables to test for the effects of these parameters on each. The *nlme* library from the R statistical programming language (R version 4.2.1) was used for the analysis using the *lme* function [24]. The subject was modeled as a random effect.

The cancerous and non-cancerous HNSCC tissue diagnosis and measurement number were fixed effects. Analysis of variance (ANOVA) was used to examine inter-group variances between normal and HNSCCs from different tissue groups. This enabled identification of the optical features that showed the statistically most significant differences between HNSCC and distant normal tissues.

## 3. Results

### 3.1. Hemoglobin Saturation Significantly Lower in Cancerous Tissues

The clinical study enrolled nineteen patients to evaluate the pressure-sensing probe and self-autocalibration for use with diffuse reflectance spectroscopy. This study tested the hypothesis that DRS could detect the altered physiology and structure of cancer compared to normal tissue [7]. We also hypothesized that the integration of self-calibration and pressure-sensing would lead to consistent and reproducible measurements.

Mean concentration for Hb saturation, total Hb and the reduced scattering coefficient *µ_s_*′ were calculated via the inverse Monte Carlo analysis algorithm, which attempts to fit the diffuse reflectance spectra to a model incorporating the known absorption and scattering properties of the tissue. This was then analyzed using the linear mixed effects model to determine if there were any significant differences between cancerous and non-cancerous tissues. Figure 2 shows these data presented as a box plot, for cancerous and non-cancerous (normal + benign) tissue types. Hb saturation in cancerous tissues was significantly lower than in non-cancerous tissues (*p* < 0.001) (Figure 2b). However, in the total hemoglobin and mean reduced scattered (*µ_s_*′), no significant differences were observed (Figure 2a,c). Also shown are examples of cancerous (Figure 2d) and normal (Figure 2e) tissue spectra and fits.

### 3.2. Effects of Probe Placement upon Repeated Measurements

**Figure 3:**
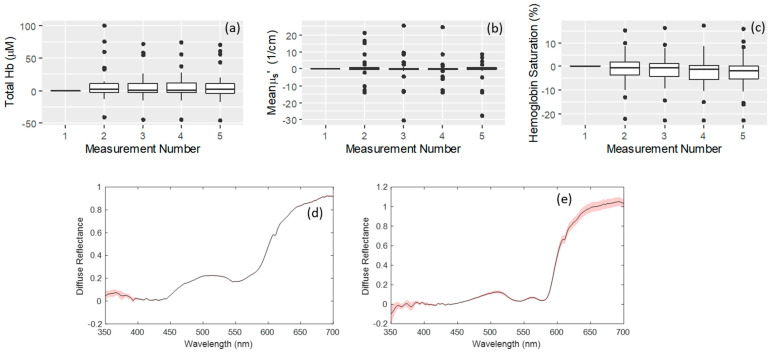
Plots showing consistency of repeated measurements. For each tissue sample, 5 repeated measurements were taken. The difference for each measurement with respect to the first measurement for that site are plotted here as box plots to indicate any trends in signal deviation. To simplify this plot, data are pooled across all measurement site types (cancer and non-cancer). For the case where there is no time dependent effect (due to probe pressure or other effect), the median value would be expected to remain at zero, indicating there is no change in signal over time. Using a linear mixed effects model indicated that there was no significant effect of the measurement repeat (*p* > 0.1), indicating that the measurements are stable over time. Difference in total Hb (**a**), Mean µs’ (**b**), and hemoglobin saturation (**c**) over five consecutive measurements are numbered 1 through 5 as indicated on the x-axis. The consistency with which individual spectra can be acquired is also illustrated in a representative example case, shown for the same tumor and normal tissue samples shown in Figure 2d,e. The mean and standard deviation of the tumor and normal spectra are plotted in (**d**,**e**), respectively, with the mean shown as a black line, and the standard deviation shown as a red outline (mainly visible at the upper and lower ends of the spectra).

Five repeated measurements were taken for each site. Neither the total hemoglobin (Figure 3a), the mean reduced scattering coefficient (Figure 3b), nor the hemoglobin saturation (Figure 3c) showed a significant difference over time as tested by a linear mixed effects model (*p* > 0.1). These results are pooled across all tissue classifications. In addition, an example of the mean spectra with standard deviation indicated are shown for an example of cancerous (Figure 3d) and normal (Figure 3e) samples.

## 4. Discussion

In this HNSCC clinical study, we assessed tumor lesions via DRS and validated the pressure-sensing and automatic self-calibration probe for reproducible measurements. The automated pressure-sensing element with software adjustments addressed two important challenges of using a DRS system for physiologic measurements: (1) the influence of uncontrolled pressure on the physiologic status of the tissues being interrogated and (2) the temporal dependence of these physiologic parameters following initiation of probe contact.

The former issue involves actually changing the tissue environment by pressing too hard with the sensor. Reif, et al. reported a study in which reflectance measurements were obtained in vivo from mouse thigh muscles while varying the contact pressure of the fiber-optic probe [25]. They found that the extracted blood vessel radius, oxygenation and Mie theory slope decreased with pressure, while the reduced scattering coefficient at 700 nm increased as a function of pressure. There are additional indications that these changes induced by probe pressure vary by tissue type [16]. Since we are attempting to reproducibly and accurately differentiate between tumor and normal tissue, it is critical that we include and validate a pressure sensor within the DRS system. Previously, we demonstrated that at higher probe-tissue contact pressures, significant alterations in optical parameters are seen in normal tissue, in particular with total hemoglobin decreasing with increasing pressure [5]. These results were used to guide selection of probe pressure to minimize the impact of probe pressure upon the measurement. This study demonstrated no significant effect due to repeated measurements (*p* > 0.1 by linear mixed effects model). With this validation, any differences between the extracted parameters are due to the functional difference between normal and cancerous tissue rather than time-dependent probe pressure effects under these experimental conditions.

Having established an appropriate, non-perturbative measurement device and technique, we sought to investigate HSNCC optical properties and identify potential diagnostic biomarkers. Significant differences in tissue hemoglobin saturation exist between cancerous and non-cancerous tissues; this has been seen in breast [26], rectum/anus [27] and head and neck cancer [28]. It is well-accepted that most solid tumors are both chronically and cyclically hypoxic due to the increase in oxygen demand and deficiencies in tumor vasculature [20]. In Figure 2, we identified a significant decrease (*p* < 0.001) in hemoglobin saturation in cancer compared to normal tissue. Additionally, there is a decreasing trend in the scattering coefficient between cancer and non-cancer. Although more samples are necessary to establish diagnostic accuracy, these results indicate that DRS may serve as a diagnostic tool intraoperatively. The difference in hemoglobin saturation between normal and cancerous tissue also highlights the importance of adequate pressure control as hemoglobin saturation is one of the most sensitive endpoints with respect to pressure.

In this study, the diffuse reflectance was fit over the spectral range of 500–600 nm. Because our primary goals in this study were (1) to utilize hemoglobin contrast to identify the expected differences in tumor hypoxia, (2) to monitor any potential perturbation in total hemoglobin concentration due to pressure and (3) to verify our ability to measure these properties consistently, we chose to use this range to provide greater weighting and optimal fitting within the 500–600 nm wavelength range, where hemoglobin shows the greatest contrast between oxygenated and deoxygenated hemoglobin; although, this may sacrifice accuracy in determination of the tissue scattering properties. This may not be the optimal wavelength range for diagnostic accuracy, and future studies could evaluate these tradeoffs, but for this purpose, this approach was found to provide consistently high quality fits over this wavelength range and so it was suitable for the needs of this study.

This study looked at a relatively small number of patients, in order to meet its goal of ensuring that the probe is calibrated properly for future, more rigorous studies that might demonstrate how functional differences between tumor and normal tissue can be used to diagnose, stage, or predict treatment response. Additional endpoints are also possible, such as the difference in parameters between tumor vs. normal sites, which could help to control for interpatient variability. The advantage of using the absolute parameters, rather than differences, is that endpoints are directly comparable and physiologically meaningful, as for example, quantification of tumor hypoxia and its potential effect on radiation therapy efficacy is likely to depend on absolute hypoxia levels, as opposed to the difference from normal. A larger study incorporating a multivariate diagnostic algorithm that can utilize multiple such endpoints, such as a linear discriminant algorithm, logistic regression, support vector machine, or other similar approach, coupled with a validation approach, is needed to fully evaluate the diagnostic potential of this tool. This is a goal of a future study.

## 5. Conclusions

In conclusion, there was minimal deviation over time for repeated measurements, indicating that automating the pressure with a sensor is successful. The automated triggering of a measurement at a set pressure is a critical attribute, particularly when the physiologic endpoints change frequently within a small window of time. We found significant differences in hemoglobin saturation between cancer and normal tissue, supporting DRS as a diagnostic tool that could minimize the number of invasive biopsies. Hemoglobin saturation is one of the most sensitive physiologic endpoints (in terms of pressure changes), and it has been identified as an important diagnostic endpoint. It was found that DRS using self-calibration and pressure sensing was able to 1) verify repeated signal stability and 2) identify changes in hemoglobin saturation. This lends confidence in this DRS tool to provide clinically useful tumor physiologic endpoints.

## Figures and Tables

**Table 1:** Summary of the histopathologic diagnosis of the tissue samples used for spectroscopic analysis.

**Study Summary**	
**Subjects in Study**	19
Subjects with cancer sites sampled	12
Subjects with normal sites sampled	14
Subjects with benign sites samples	1
**Specifics of Sampled Sites**	
**Squamous Cell Carcinoma**	Benign
8 tongue 1 larynx 1 oropharynx 1 tonsil 1 piriform lesion	1 benign atypical verrucous hyperplasia

## Data Availability

All data are available upon request.
